# Comparison of Consecutive Therapeutic Effects of Nanoemulsion and Emulsion Cyclosporin in Dry Eye Patients after Short-Term Treatment with Topical Fluorometholone

**DOI:** 10.1155/2022/6037401

**Published:** 2022-02-09

**Authors:** Yeon Sun Choi, Hae Jung Paik, Dong Hyun Kim

**Affiliations:** Department of Ophthalmology, Gil Medical Center, Gachon University College of Medicine, Incheon, Republic of Korea

## Abstract

**Purpose:**

To compare the consecutive therapeutic effects of 0.05% emulsion and nanoemulsion cyclosporine (CsA) in dry eye patients after short-term treatment with unpreserved 0.1% fluorometholone (FML).

**Methods:**

A prospective, randomized, and double-blinded study of dry eye patients was conducted in a single center. Patients were assigned to the nanoemulsion CsA (group 1) and emulsion CsA (group 2) groups. To relieve discomfort, unpreserved 0.1% FML was used in both groups for 4 weeks and then changed to 0.05% CsA for the next 8 weeks. Symptom assessment in dry eye (SANDE) score, tear secretion, tear film breakup time (TBUT), corneal staining score (CSS), meibomian gland dysfunction (MGD) grade, and meibomian gland (MG) expression were evaluated at baseline and at 4 and 12 weeks after treatment.

**Results:**

Twenty-four patients completed the treatment (9 and 15 patients in groups 1 and 2); in both the groups, SANDE score, TBUT, MGD grade, and MG expression were significantly improved after treatment with unpreserved 0.1% FML (each *p* < 0.005), and the therapeutic effects were enhanced with changes in nanoemulsion or emulsion CsA compared with baseline (each *p* < 0.001). TBUT and CSS after treatment in group 1 were significantly improved compared to those in group 2 (*p*=0.003 and 0.020, respectively).

**Conclusion:**

Consecutive therapeutic effects of nanoemulsion or emulsion CsA after short-term treatment with unpreserved FML were excellent in patients with dry eyes. Topical nanoemulsion CsA showed better improvement in TBUT and OSS than CsA. This trial is registered with KCT0006070.

## 1. Introduction

According to the TFOS DEWS II, dry eye disease (DED) is a multifactorial disease of the ocular surface characterized by a loss of homeostasis of the tear film accompanied by ocular symptoms in which tear film instability and hyperosmolarity, ocular surface inflammation and damage, and neurosensory abnormalities play etiological roles [1–3]. In addition, meibomian gland dysfunction is known as a representative cause of DED because it induces tear film abnormality and instability [4, 5].

Cyclosporine (CsA) is a lipophilic peptide composed of 11 amino acids that acts as a calcineurin inhibitor to prevent the infiltration of T cells and suppress several inflammatory cytokines. CsA binds to cyclophilin in the cytoplasm of T cells to form a CsA/cyclophilin complex that blocks calcineurin-mediated dephosphorylation of nuclear factor of activated T cells and interrupts the transcription of cytokines, including IL-2 and IL-4. Inhibition of IL-2 formation blocks T-cell proliferation and suppresses T-cell-mediated immune responses [6, 7]. In this manner, topical CsA is known to reduce the activated lymphocytes of the conjunctiva, inhibit the factors associated with inflammation and cell death, and increase the density of goblet cells in the conjunctiva; thus, it is used as a therapeutic agent for dry eye patients to improve tear production [5, 6]. Byun et al. reported that 0.05% CsA eye drops improved the clinical symptoms of dry eye patients, such as foreign body sensation, blurred vision, photosensitivity, and pain [8]. In another study, 0.05% CsA increased tear secretion and improved subjective symptoms in chronic DED patients [9]. Chang et al. reported that 0.05% CsA with 0.1% hyaluronic acid (HA) improved the tear breakup time (TBUT) and tear osmolarity [10]. Meanwhile, topical corticosteroids are generally indicated for the treatment of ocular inflammatory diseases because they can inhibit proinflammatory cytokines and chemokines, stabilize macrophages and neutrophils, and repress the key enzymes involved in the initiation or maintenance of the inflammatory response [11]. Short-term topical 0.1% fluorometholone showed excellent therapeutic effects in patients with refractory DED or acute DED flares in our previous study [12].

Since CsA is insoluble in water, the 0.05% CsA eye drop (Restasis®, Allergan Inc., CA, USA) is in the form of an emulsion, with castor oil added to increase its solubility [13]. A well-known side effect of 0.05% CsA emulsion is a burning sensation, which decreases patient compliance [13]. In order to minimize this adverse effect, a 0.05% CsA in nanoemulsion form was developed. The nanoemulsion CsA eye drops were transparent, and their particle sizes were uniform. However, the therapeutic effects of emulsion and nanoemulsion CsA have not been fully investigated. Thus, we compared the consecutive therapeutic effects of 0.05% nanoemulsion and emulsion CsA after common short-term treatment with unpreserved fluorometholone (FML) in DED patients.

## 2. Materials and Methods

This study was approved by the Institutional Review Board (approval number GBIRB2016-238) according to the Declaration of Helsinki and was registered in the Clinical Research Information Service (https://cris.nih.go.kr). A prospective, randomized, and double-blinded study of dry eye patients was conducted in a single center, and 50 patients were enrolled from May 2018 to October 2019.

Patients older than 19 years who visited our clinic with complaints such as ocular dryness, burning, and hyperemia were examined to rule out DED, and we performed Schirmer's test without anesthesia and assessed tear film breakup time (TBUT) using standardized procedures. DED was diagnosed according to the diagnostic guidelines of the Korean Corneal Disease Study Group and TFOS II [1, 2]. Those with Schirmer's test outcome of <10 mm at the end of 5 min or a TBUT of <10 s participated in this study. Patients with a history of Sjogren's syndrome or who were taking systemic steroids or immunosuppressive agents, contact lens wearers, or patients who had DED induced by chronic drug usage, such as antidepressants and antihistamines, were excluded. Patients who had prior ocular surgery within 3 months or were on any other eye drops, such as lubricants or antiglaucoma medications, were also excluded.

Patients were randomly assigned to one of the two groups, nanoemulsion CsA (T-sporin^®^, Hanlim Pharm Co., Ltd., Seoul, Korea) group or emulsion CsA (Restasis^®^, Allergan Inc., Irvine, CA, USA) group using the block randomization method (groups 1 and 2). For rapid symptom relief, all patients in groups 1 and 2 were treated with unpreserved 0.1% FML (Fumelon^®^, Hanlim Pharm Co., Ltd., Seoul, Korea) four times a day for the first 4 weeks, and then, they were changed to 0.05% emulsion or nanoemulsion CsA twice a day for the next 8 weeks. All patients used 0.15% hyaluronic acid artificial tears (Hyaluronmax^®^, Hanlim Pharm Co., Ltd., Seoul, Korea) four times a day for 12 weeks. The type of allocated drug (nanoemulsion or emulsion CsA) was masked to the examiner (DH Kim) and patients.

At baseline and 4 and 12 weeks after treatment, best-corrected visual acuity (BCVA), intraocular pressure, symptom assessment in dry eye (SANDE) score, TBUT, Schirmer's test, corneal staining score (CSS, National Eye Institute Scale, 0–15), MGD grade (0–4), and MG expression (0–3) were examined to compare the therapeutic effects between the two groups [7, 14–16].

Statistical analysis was performed using SPSS program version 22.0 (IBM Corp., Armonk, NY, USA). Data are presented as mean ± standard deviation (SD). The Wilcoxon signed-rank test and Mann–Whitney *U* test were used for intragroup and intergroup analyses. Differences were considered statistically significant at *p* < 0.05.

## 3. Results

**Figure 1:**
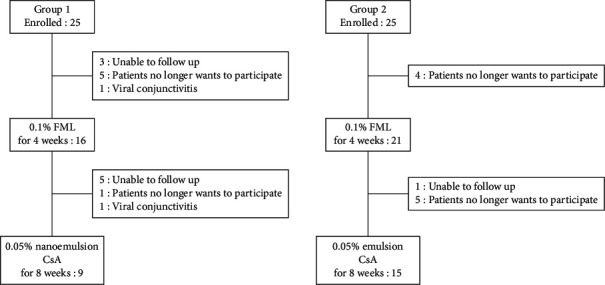
Schematic illustration of enrolled patients.

A total of 50 patients (12 men and 38 women) were enrolled in the study, and the mean age of the patients was 61.2 ± 9.1 years. At the 12-week follow-up, 9 patients (4 men and 5 women) in group 1 and 15 patients (4 men and 11 women) in group 2 completed this clinical trial. Thirteen patients were excluded at 4 and 12 weeks (Figure 1). Fifteen patients no longer wanted to participate in this trial, nine patients were unable to be contacted, and two patients were excluded from the clinical trial because of acute viral conjunctivitis (Figure 1). The mean age of patients in groups 1 and 2 was 58.6 ± 11.4 and 62.1 ± 6.4 years, respectively (*p*=0.279). The best-corrected visual acuity (BCVA), intraocular pressure, MGD grade, and MG expression at baseline were also not significantly different between the two groups (*p* > 0.05) (Table 1).

In groups 1 and 2, SANDE score, TBUT, MGD grade, and MG expression were significantly improved after treatment with unpreserved 0.1% FML for the initial 4 weeks (each *p* < 0.005, Table 2) and intraocular pressure did not increase significantly (*p*=0.873/0.723, Table 2). CSS did not significantly change before and after 0.1% FML treatment (group 1/2: *p*=0.408/0.750, Table 2). There was no significant increase in tear secretion in group 1, but there was a slight decrease in tear secretion in group 2 (group 1/2: *p*=0.396/0.008, Table 2). After 4 weeks of FML treatment, BCVA, SANDE score, TBUT, CSS, MGD grade, and MG expression were not significantly different between groups 1 and 2 (each *p* > 0.05) but the tear secretion was reduced in group 2 compared to that in group 1 (*p*=0.037, Table 2).

After consecutive treatment with 0.05% CsA for the next 8 weeks (12 weeks from baseline), both groups showed significant improvement in SANDE score, TBUT, MGD grade, and MG expression compared to baseline (each *p* < 0.001, Table 3). CSS showed no differences compared to baseline in both groups (group 1/2: *p*=0.563/0.750, Table 3), and BCVA and intraocular pressure also showed no specific changes (each *p* > 0.05). There was some decrease in tear secretion in group 2 compared to baseline, but it was not statistically significant (*p*=0.070, Table 3).

**Figure 2:**
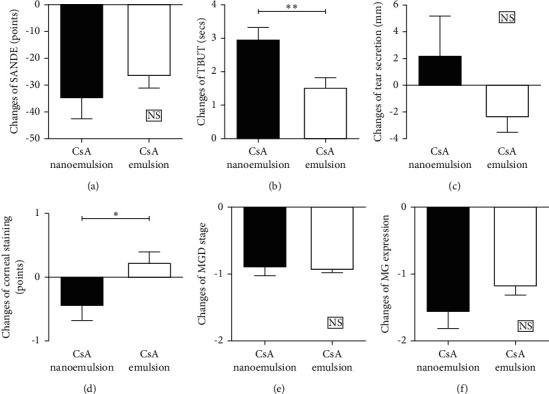
Comparison of changes in dry eye parameters between nanoemulsion and emulsion CsA groups at 12 weeks after treatment with initial topical steroid and consecutive topical CsA compared to baseline. CsA, cyclosporin; SANDE, symptom assessment in dry eye; TBUT, tear film breakup time; MGD, meibomian gland dysfunction; MG, meibomian gland.

We compared the changes in SANDE score, TBUT, tear secretion, MGD grade, and MG expression from baseline between groups 1 and 2 at the time point of 12 weeks after treatment. TBUT and CSS were significantly improved in group 1 compared to those in group 2 (TBUT/CSS: *p*=0.003/0.020, Figure 2, Mann–Whitney *U* test). However, there were no differences in SANDE score, tear secretion, MGD grade, or MG expression between the two groups (each *p* > 0.05, Figure 2, Mann–Whitney *U* test).

**Figure 3:**
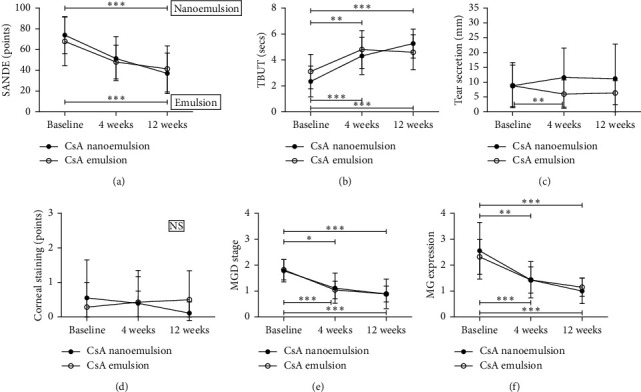
Changes in dry eye parameters at baseline and 4 and 12 weeks after treatment. CsA, cyclosporin; SANDE, symptom assessment in dry eye; TBUT, tear film breakup time; MGD, meibomian gland dysfunction; MG, meibomian gland.

Figure 3 shows the overall changes in SANDE score, TBUT, tear secretion, CSS, MGD grade, and MG expression at baseline and at 4 and 12 weeks after treatment between groups 1 and 2. In both groups, there were meaningful improvements in SANDE score, TBUT, MGD grade, and MG expression at 4 weeks after treatment with FML compared to baseline and therapeutic effects were enhanced after treatment with nanoemulsion or emulsion CsA for an additional 8 weeks (each *p* < 0.001). Both groups showed no differences in tear secretion and corneal staining compared to the baseline (each *p* > 0.05).

## 4. Discussion

In this prospective, randomized, double-blinded study, unpreserved 0.1% FML for 4 weeks and consecutive 0.05% nanoemulsion or emulsion CsA for the next 8 weeks showed excellent therapeutic effects in patients with DED. SANDE score, TBUT, MGD grade, and MG expression were significantly improved after treatment with 0.1% FML, and these therapeutic effects were greatly enhanced with changes in nanoemulsion or emulsion CsA. TBUT and CSS were more improved in the nanoemulsion CsA group than in the emulsion CsA group.

CsA is a lipophilic material that is separated from the fungus *Tolypocladium inflatum* and has the immunosuppressive function of inhibiting IL-2 to suppress T-cell proliferation [5, 7]. Systemic CsA has been used to prevent rejection of organ transplantation [5]. In the ophthalmic area, topical CsA has been used to prevent corneal graft rejection through immune suppression or to modulate inflammatory ocular surface diseases such as vernal keratoconjunctivitis, ocular GVHD, and Mooren's ulcer [5, 7, 8]. Topical CsA has been widely used, especially in patients with DED, since the emulsion type of CsA (Restasis®, Allergan Inc., Irvine, CA, USA) was released about 20 years ago. The molecules of CsA have a very hard structure due to hydrogen bonds associated with the ring structure due to C62H111N11O12; thus, the solubility in water is very low [13]. Olive oil or corn oil can be expected to induce better ocular penetration, but Williams reported that CsA, which is delivered in olive oil solution, is reported to cause a burning sensation in the conjunctiva [13]. Byun et al. reported that 72% of DED patients treated with CsA were satisfied and that the symptoms of DED and tear secretion were much improved, but there were definite side effects, including an irritating sensation after administration [8].

**Figure 4:**
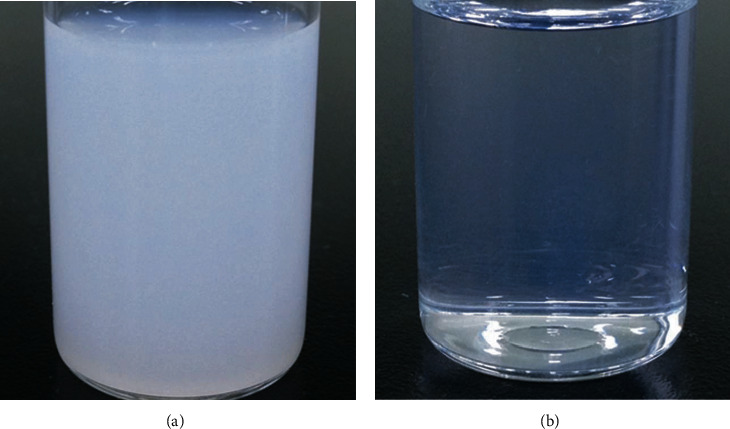
Comparison of transparency between emulsion and nanoemulsion CsA. (a) Emulsion CsA. (b) Nanoemulsion CsA. CsA, cyclosporin.

An emulsion is a liquid-liquid dispersion technology in which one or more liquids are dispersed in other liquids that are not mixed. It is an effective topical ophthalmic drug delivery method, especially oil-water emulsions, which are known to be effective in delivering lipophilic drugs. Owing to the recent development of a new emulsifier, emulsion technology is facing a new turning point in terms of the preparation of CsA [17, 18]. CsA has a large molecular weight, and its affinity for water is very low, making it difficult to use an effective agent as an existing ophthalmic drug delivery system. Therefore, various drug delivery technologies, such as hydrogels, in situ gelling systems, liposomes, nanoparticles, and micelles, have been developed [17]. The Restasis® eye drops (Allergan Inc., Irvine, CA, USA) are an anionic oil-in-water emulsion without a preservative; CsA is dissolved in castor oil with an emulsifier, polysorbate [18]. However, as Restasis® is an anisotropic complex emulsion, when it comes in contact with the tear film, the surfactant may be released. In addition, there could be creamization or aggregation because of the uneven size of CsA particles, so the physical and chemical long-term stability is reduced. Therefore, side effects such as blurred vision, conjunctival congestion, and burning sensations after instillation have been frequently reported [19–21]. In addition, CsA is present in various phases, so that it cannot be sufficiently delivered to the tissue [19]. Meanwhile, Cyporin-N^®^ (CsA, Cyporin-N^®^, Taejoon Pharm Co., Seoul, Korea) or 0.05% T-sporin^®^ (CsA, T-sporin^®^, Hanlim Pharm Co., Ltd., Seoul, Korea) are nanoemulsion CsA products based on a self-nanoemulsifying drug delivery system (SNEDDS) method [22–25]. SNEDDS is an anhydrous homogenous mixture made by mixing oil, drugs, surfactants, and auxiliary surfactants. Its particle size is very small and even. As a transparent nanoemulsion in a nanometer unit, it is known to have excellent chemical and physical stability [22–25] (Figure 4).

Kim et al. reported that the nanoemulsion CsA was more effective in improving conjunctival damage and tear film stability than the emulsion CsA in patients with DED [22]. Shin et al. reported that the OSDI score and foreign body sensation were lower in the nanoemulsion CsA group, and both the nanoemulsion and emulsion CsA groups showed similar therapeutic effects in objective parameters of DED after 12 weeks of treatment [26]. In contrast with previous studies, this study showed significant improvement in MGD, which can be a major risk factor for DED. In addition, after the initial short-term FML treatment, we quickly alleviated the irritating symptoms of DED and then switched to the CsA protocol to continuously maintain ocular comfort and improve objective DED parameters. In particular, the nanoemulsion CsA group (group 1) showed better TBUT and CSS than the emulsion CsA group (group 2), even though there were slight differences.

Rye et al. showed that short-term corticosteroids can effectively improve objective findings and subjective symptoms in patients with acute exacerbation of DED or refractory DED [12]. One hundred thirty-seven DED patients treated with topical steroids for 4 weeks showed a significant improvement in SANDE score, TBUT, ocular surface staining, and MGD grade [12]. In particular, those effects were superior in tear MMP-9-positive patients [12]. CsA can also improve the symptoms and signs of DED, but improvement takes more than 2-3 months [12, 27]. Since up to 17% of patients complain of burning sensation, there are numerous clinical interventions to quickly improve the acute exacerbation of DED [28–31]. Sheppard et al. showed that two weeks of topical steroid usage before starting long-term topical CsA in chronic DED patients resulted in a rapid and effective decrease in the symptoms and signs of DED [32]. Also, prior use of a topical steroid reduces the burning sensation and discomfort due to CsA eye drops, resulting in an improvement in patient compliance so that the inflammatory response can effectively introduce CsA onto the ocular surface [32]. Byun et al. reported that the short-term use of topical steroid allows fast symptom relief in moderate-to-severe dry eye patients without any severe complications [33]. Singla et al. also reported that the combination of topical steroids and CsA had a better treatment effect than CsA single therapy in patients with moderate dry eye syndrome [34]. Therefore, rapid symptom relief with short-term topical steroids and then switching to CsA could be a good treatment option to gain clinical efficiency and safety in DED patients with severe symptoms.

This study has several limitations. There were many drop-out patients, and the sample size was small. Quantitative analysis of reduced inflammation in the tear film and ocular surface was not performed. There were more drop-out patients in the nanoemulsion group than in the emulsion group, and it is necessary to analyze the cause, such as irritating sensation or discomfort in usage. However, we found that consecutive CsA treatment following short-term topical steroid administration rapidly improved and maintained long-term enhancement in DED symptoms and signs as well as MGD. In addition, more extensive studies are required to determine whether nanoemulsion CsA is superior to emulsion CsA.

## 5. Conclusions

In conclusion, the consecutive therapeutic effects of CsA after short-term, unpreserved topical steroid use were clinically excellent in DED patients. Topical nanoemulsion CsA showed better improvement in TBUT and OSS than emulsion CsA; hence, nanoemulsion CsA may be more helpful in DED patients.

## Figures and Tables

**Table 1:** Baseline characteristics in patients.

	Group 1 (*n* = 9) (nanoemulsion CsA)	Group 2 (*n* = 15) (emulsion CsA)	*p* value
Age (years)^†^	58.6 ± 11.4	62.1 ± 6.4	0.279
Sex (Male/female)^‡^	4/5	4/11	0.355
BCVA (logMAR)^†^	0.08 ± 0.11	0.12 ± 0.11	0.262
IOP (mmHg)^†^	9.4 ± 1.6	9.8 ± 2.4	0.643
SANDE score^†^	73.9 ± 17.8	67.8 ± 24.1	0.344
TBUT (sec)^†^	2.6 ± 1.1	2.3 ± 0.7	0.403
Schirmer (mm)^†^	9.0 ± 7.6	8.9 ± 7.2	0.077
CSS (pts)^†^	0.6 ± 1.1	0.3 ± 0.7	0.248
MGD stage (0–4)^†^	1.8 ± 0.4	1.9 ± 0.4	0.574
MG expression (pts)^†^	2.6 ± 1.1	2.3 ± 0.7	0.360

Values are presented as the mean ± standard deviation. ^†^Mann–Whitney *U* test; ^‡^the chi-square test. CsA: cyclosporin; BCVA: best corrected visual acuity; IOP: intraocular pressure; SANDE: symptom assessment in dry eye; TBUT: tear film breakup time; CSS: corneal staining score (NEI scale, 0–15); MGD: meibomian gland dysfunction; MG: meibomian gland.

**Table 2:** Comparison of therapeutic effects at 4 weeks after treatment with topical FML between nanoemulsion and emulsion CsA groups.

	Group 1 (nanoemulsion CsA)			Group 2 (emulsion CsA)			*p* value^‡^
	Baseline	4 weeks after treatment	*p* value^†^	Baseline	4 weeks after treatment	*p* value^†^	
BCVA (logMAR)	0.08 ± 0.11	0.06 ± 0.07	0.348	0.12 ± 0.11	0.10 ± 0.10	0.407	0.132
IOP (mmHg)	9.4 ± 1.6	9.6 ± 1.4	0.873	9.8 ± 2.4	9.6 ± 2.1	0.723	0.731
SANDE (pts)	73.9 ± 17.8	51.2 ± 21.3	0.002^*∗*^	67.8 ± 24.1	49.4 ± 15.8	<0.001^*∗*^	0.588
TBUT (sec)	2.6 ± 1.1	4.3 ± 1.5	0.001^*∗*^	3.1 ± 1.4	4.8 ± 1.5	<0.001^*∗*^	0.296
Schirmer (mm)	9.0 ± 7.6	11.6 ± 10.0	0.396	8.9 ± 7.2	6.1 ± 4.9	0.008^*∗*^	0.037
CSS (pts)	0.6 ± 1.1	0.4 ± 0.8	0.408	0.3 ± 0.7	0.4 ± 0.9	0.750	0.999
MGD grade	1.8 ± 0.4	1.1 ± 0.6	0.001^*∗*^	1.9 ± 0.4	1.0 ± 0.3	<0.001^*∗*^	0.536
MG expression (pts)	2.6 ± 1.1	1.4 ± 0.7	0.001^*∗*^	2.3 ± 0.7	1.4 ± 0.5	<0.001^*∗*^	0.752

Values are presented as mean ± standard deviation. ^†^Intragroup analysis (Wilcoxon signed-rank test); ^‡^intergroup analysis (Mann–Whitney *U* test). FML: 0.1% fluorometholone; BCVA: best corrected visual acuity; IOP: intraocular pressure; SANDE: symptom assessment in dry eye; TBUT: tear film breakup time; CSS: corneal staining score (NEI scale, 0–15) MGD: meibomian gland dysfunction; MG: meibomian gland. ^*∗*^Statistically significant.

**Table 3:** Comparison of therapeutic effects at 12 weeks after treatment with topical FML and CsA between nanoemulsion and emulsion CsA groups.

	Group 1 (nanoemulsion CsA)			Group 2 (emulsion CsA)			*p* value^‡^
	Baseline	12 weeks after treatment	*p* value^†^	Baseline	12 weeks after treatment	*p* value^†^	
BCVA (logMAR)	0.08 ± 0.11	0.06 ± 0.11	0.390	0.12 ± 0.11	0.10 ± 0.11	0.407	0.264
IOP (mmHg)	9.4 ± 1.6	9.2 ± 1.4	0.888	9.8 ± 2.4	9.3 ± 2.7	0.723	0.639
SANDE score	73.9 ± 17.8	37.0 ± 19.7	<0.001^*∗*^	67.8 ± 24.1	41.3 ± 23.0	<0.001^*∗*^	0.803
TBUT (sec)	2.6 ± 1.1	5.3 ± 1.1	<0.001^*∗*^	3.1 ± 1.4	4.6 ± 1.4	<0.001^*∗*^	0.037^*∗*^
Schirmer (mm)	9.0 ± 7.6	11.1 ± 11.6	0.418	8.9 ± 7.2	6.7 ± 4.0	0.070	0.622
CSS (pts)	0.6 ± 1.1	0.1 ± 0.3	0.563	0.3 ± 0.7	0.5 ± 0.8	0.750	0.118
MGD grade	1.8 ± 0.4	0.9 ± 0.6	<0.001^*∗*^	1.9 ± 0.4	0.9 ± 0.3	<0.001^*∗*^	0.896
MG expression (pts)	2.6 ± 1.1	1.0 ± 0.5	<0.001^*∗*^	2.3 ± 0.7	1.2 ± 0.4	<0.001^*∗*^	0.274

Values are presented as the mean ± standard deviation. ^†^ Intragroup analysis (the Wilcoxon signed-rank test); ^‡^intergroup analysis (Mann–Whitney *U* test). FML, 0.1% fluorometholone; CsA, 0.05% cyclosporine; BCVA: best corrected visual acuity; IOP, intraocular pressure; SANDE, symptom assessment in dry eye; TBUT, tear film breakup time; CSS, corneal staining score (NEI scale, 0–15); MGD, meibomian gland dysfunction; MG, meibomian gland. ^*∗*^Statistically significant.

## Data Availability

The data that support the findings of this study are available from the corresponding author upon reasonable request.
